# Tumor and the Microenvironment: A Chance to Reframe the Paradigm of Carcinogenesis?

**DOI:** 10.1155/2014/934038

**Published:** 2014-06-12

**Authors:** Mariano Bizzarri, Alessandra Cucina

**Affiliations:** ^1^Department of Experimental Medicine, “Sapienza” University of Rome, Systems Biology Group, Viale Regina Elena 324, Via A. Scarpa 14, 00161 Rome, Italy; ^2^Department of Surgery “Pietro Valdoni”, “Sapienza” University of Rome, Via A. Scarpa 14, 00161 Rome, Italy

## Abstract

The somatic mutation theory of carcinogenesis has eventually accumulated an impressive body of shortfalls and paradoxes, as admittedly claimed by its own supporters given that the cell-based approach can hardly explain the emergence of tissue-based processes, like cancer. However, experimental data and alternatives theories developed during the last decades may actually provide a new framework on which cancer research should be reframed. Such issue may be fulfilled embracing new theoretical perspectives, taking the cells-microenvironment interplay as the privileged level of observation and assuming radically different premises as well as new methodological frameworks. Within that perspective, the tumor microenvironment cannot be merely considered akin to new “factor” to be added to an already long list of “signaling factors”; microenvironment represents the physical-biochemical support of the morphogenetic field which drives epithelial cells towards differentiation and phenotype transformation, according to rules understandable only by means of a systems biology approach. That endeavour entails three fundamental aspects: general biological premises, the level of observation (i.e., the systems to which we are looking for), and the principles of biological organization that would help in integrating and understanding experimental data.

## 1. Introduction: From the “Cancer Hallmarks” to the “Shortfalls” of the Somatic Mutation Theory of Carcinogenesis

Cancer is commonly thought as the result of progressive accumulation of random mutations and increased deregulation of key molecular pathways (somatic mutation theory of carcinogenesis, SMT) [1]. This statement utterly relies on a tacit premise that assumes that the pathogenetic process depends on alterations in a discrete number of signalling pathways, thought to carry “instructive” biological “information.” That paradigm prompted the pharmaceutical industry to focus on development of drugs targeting specific molecular components, funding basic research, and technological applications in line with those premises, thus strengthening a bottom-up reductionist approach. Among others, Hanahan and Weinberg have been of the most prominent standard-bearers of the mainstream and they have substantially contributed in popularizing the SMT among scientists [2]. However, recently Weinberg called into question the entire theoretical construct of SMT. Quoting him, “half a century of cancer research had generated an enormous body of observations [⋯] but there were essentially* no insights into how the disease begins and progresses*” [3]. Despite the expectations raised by “the Ame's axiom (“substances act as carcinogens because they have mutagenic activity”), it shortly turned out that most powerful carcinogens are actually not mutagen”; “but fortunately—as Weinberg candidly admits—*I and others were not derailed by discrepant facts.*” Indeed, a whole series of “discrepant facts” and many other paradoxes were ignored or marginalized, while acknowledging that their realistic appraisal would have flawed the dominant paradigm [4]. Nonetheless, the search for mutated oncogenes and/or tumor suppressor continued unabated up to the present. “But even this was an* illusion*, as only became apparent years later [⋯] the identities of mutant cancer-causing genes varied dramatically from one type of tumor to the next [⋯]. Each tumor seemed to represent a unique experiment of nature.” Rather unexpectedly, however, Weinberg concludes that, at the beginning of 2014, “we cannot really assimilate and interpret most of the data that we accumulate. How will all this play out? I wouldn't pretend to know. It's a job [⋯] for the next generation. Passing the buck like this is an enormously liberating experience.”

## 2. Thinking of Cancer as a Tissue-Based Disease

The core message of Weinberg's admissions is that current SMT-based cancer research provided no meaningful results to solve the cancer puzzle because it lacks a well-founded and robust theory of biological phenomena. According to Weinberg, “we lack the conceptual paradigms and computational strategies for dealing with this complexity. And equally painful, we do not know how to integrate individual data sets, such as those deriving from cancer genome analyses, with other equally important data sets, such as proteomics.” Moreover, that unfortunate situation must still be investigated even more thoroughly, and the “abscess” should be incised widely if we want to get the healing. Indeed, the confusion and contradictions featuring the current state of cancer research can be ascribed to several factors, among which the absence of a true biological theory, inappropriate level on which experiments and observations are made, and associated methodological biases [5]. Biology is indeed facing a dilemma the physics has solved a century ago, that is, how to cope with an overwhelming body of data without having a general theoretical framework into which experimental results could be embedded [6]. In other words, we need to recognize general organizing principles that could enable us to frame a reliable theoretical structure to which the experimental work will be inextricably intertwined [7]. This does not imply that in biology there is no theory at all. No science can be built up in the absence of theoretical constructs. As an example, evolutionary biology offers a general theoretical structure. However, evolution is no more than a general framework within which biological processes are interpreted, for evolution does not allow specific predictions in the way quantum mechanics does in physics. Moreover, the theory of evolution itself is ongoing reinterpretations in the light of some “unexpected” and apparently contradictory data (i.e., the inheritance of acquired characters) [8, 9]. Also, during the last four decades, biology has been grounded on a reductionistic framework [10], based on the central dogma of the biology, which states that information flows unidirectionally (from DNA to proteins, from genotype to phenotype). Implicitly, form and functions in organism would depend solely on “genetic information.” Such assumptions have never been presented as a theory, even worse, that model was presented as an unquestionable fact. However, this model is currently encompassing several drawbacks, grounded on conflicts with the gene-centric paradigm [11]. The overall picture is by far more complex than previously thought; biological interactions that take place at lower molecular levels are characterized by unpredictable nonlinear dynamics and become tightly influenced by higher-level organizational constraints [12]. Thereby, as we are actually unable to grasp such overwhelming complexity, we are also unable to set a reliable theory of biological organization [13].

This is especially true in the field of carcinogenesis, where both experimental modelling and theoretical framework have been for a long time dominated by the SMT [14, 15]. SMT explains cancer as a process taking place at the cellular/subcellular level of biological organization and claims that cancer is a problem of regulatory control of cell proliferation and of invasiveness, due to mutations and/or overexpression of a specific class of genes. Yet, current therapeutic approaches based on that paradigm have been proven to be ineffective in clinical cancer management, and the effect of new treatments for cancer on mortality has been largely disappointing [16], to the point that an increasing number of voices are asking to revise current treatment strategies [17, 18]. Undoubtedly, SMT has fostered a significant development of molecule-based technologies, providing so far a huge body of “raw” data on gene and proteomic networks; but, on the other hand, SMT has also generated an* impasse* in cancer studies, given that an increasing number of experimental results contradict its premises [19]. In turn, a number of ad hoc variants have been proposed [20], striving to include the tumor-microenvironment paradigm within SMT, while retaining unaltered the notion that cancer is a cell-based disease [21]. However, as “the current dominant paradigm wherein multiple genetic lesions provide both the impetus for and the Achilles heel of cancer might be inadequate to understand cancer as a disease process” [22], some radical alternatives, grounded on clearly different premises and epistemological settings, have been proposed [23, 24]. Yet, it is rather unpleasant to notice that no mention of those “alternatives” is reported in the Weinberg's paper.

An alternative to SMT is tissue organization field theory (TOFT), which posits that cancer arises from the deregulated interplay among cells (cells/stroma) and their microenvironment [25]. According to TOFT, the microenvironment represents the physical-biochemical support of the morphogenetic field which drives epithelial cells towards differentiation and phenotype transformation, according to rules understandable only by means of a systems approach [26]. Not only microenvironment-cells interplay is a matter of “signalling interaction” but also it involves biophysical factors and field-based effects, usually overlooked by the current scientific mainstream [27]. The structure of determination in TOFT includes both bottom-up and top-down as well as reciprocal causation. Moreover, a profound rewiring of the theoretical assumptions on which SMT has been based is required if one would consider the tumor microenvironment as a basis for making a new paradigm in cancer research. Such rewiring entails three fundamental aspects: (a) general biological premises, (b) the level of observation (i.e., the systems to which we are looking for), and (c) the principles of biological organization that should help in integrating and understanding experimental data. Overall, these features contribute to shape a novel biological theory that is still waiting for a cogent formulation [28]. Yet, in this respect, SMT and TOFT differ significantly, because they rely on radically different theoretical assumptions [29].

## 3. Carcinogenesis Theory May Be Rebuild upon the Tumor Microenvironment Paradigm

Studies on tumor microenvironment are dating back even from 1940, when microenvironment was shown to suppress skin carcinogenesis induced by chemical carcinogens [30]. Later, even experiments done to demonstrate that a single or few mutated genes are needed to induce carcinogenesis acknowledged that microenvironmental factors were mandatorily required to promote oncogenesis at the tissue level [31, 32]. As case in point, experimental tumors obtained by inoculating cells with oncogenic virus demonstrated that the context plays a pivotal role in driving the neoplastic transformation. The tumorigenicity of polyoma virus-transformed BALB/C 3T3 cells in syngeneic mice depends on the microenvironment in which these cells were grown rather than on the content of the polyoma middle T oncogene [33]. Moreover, given that no specific genetic traits have been associated so far to the metastatic process, despite aggressive efforts to find a correlation among genome profile and cancer malignancy [34], increased attention has recently been given to the microenvironment thought to “confer” a “metastatic phenotype” [35]. This is closely linked to the “seed and soil” hypothesis, first proposed by Stephen Paget [36].

Indeed, those preliminary investigations highlighted that potent carcinogenic cues could be overridden by embryonic microenvironment [37], a finding that was recently confirmed. Namely, cancer cells cultured in an embryonic environment [38–40] or cocultured in 3D-matrices with normal human cells showed apoptosis and differentiation and eventually were reprogrammed into normal phenotypes [41, 42]. Such effects have been ascribed to undefined “signalling molecules,” to morphogens, or to soluble factors provided by the morphogenetic embryonic field. Comparable results have been obtained by culturing cancer cells in 3D-matrices with normal human cells. Indeed, a matrix containing both type I collagen and reconstituted basement membrane and the presence of normal breast fibroblasts constituted the minimal permissive microenvironment to induce near-complete tumor phenotype reversion [43].

For a while, interest on immunologic [44] and angiogenic [45] aspects of tumor microenvironment overshadowed the contribution of the microenvironment on cancer initiation [46]. Yet, it is now firmly established that the microenvironment actively contributes to initiation of carcinogenesis, given that it profoundly influences tissue organization (i.e., the very shape and structure of epithelia) as well as intracellular processes in the cells within epithelial structures, like proliferation, differentiation, and apoptosis [47, 48]. Even subtle differences, in ECM composition and stiffness, selectively foster or inhibit proliferation by modulating cell cycle regulatory molecules and early response genes [49–51]. The microenvironment regulates the transcription of genes associated with differentiating pathways [52, 53] and participates in shaping cells phenotypes by modulating cell-stroma interactions and cytoskeleton architecture [54, 55]. Moreover, cell shape and microenvironmental cues trigger programmed cell death signals, hence driving cells towards apoptosis [56, 57]. Changes in the microenvironment structure or composition frequently lead to tissue fibrosis, augmented collagen crosslink, and tissue stiffening, all of which have been associated to an increased risk of developing cancer [58, 59]. It is not trivial to recall that aging is associated to increase in both tissue stiffness and cancer incidence [60]. In turn, tissue fibrosis and modification of physicochemical properties of ECM may likely influence tumor onset and progression by regulating soluble factors involved in inflammation [61] and angiogenesis [62].

Participation of the microenvironment in carcinogenesis is further supported by phenotypic changes, observed changes in stromal cells residing within the tumor microenvironment. Both cancer-associated fibroblasts [63] and endothelial cells [64] showed indeed altered architecture [65] and paracrine signals expression that ultimately lead to malignancy [66, 67] and genetic instability of the epithelial cell layer [68]. Indeed, in a seminal paper, Maffini et al. [69] demonstrated that, by treating stroma with the chemical carcinogen N-nitroso-methylurea, stromal cells may drive malignant transformation of epithelial cells by disrupting the normal stromal-epithelial interactions, whereas treating directly the epithelial cells does not lead to any cancerous transformation. Conversely, “normalizing” the behavior of “altered” stromal components of the tumor microenvironment may reduce the malignancy phenotype of tumor cells [70]. Similar results have been confirmed by others [71, 72], thereby outlining that the main factor in driving the carcinogenic process lies on the cross-talk in the microenvironment among stroma and epithelial cells.

Such results are in overt contradiction with SMT and its “oncogene” paradigm. Moreover, phenotypic traits cannot be considered a simple, linear output coded by “genomic information.” Instead, phenotypic features result from the complex cross-talk among three-dimensional participants within the microenvironment. Additionally, the regulatory control exerted by the microenvironment on the emergence of tumor clones contributes to explain why “occult” tumors are prevented from progressing into overt, clinical cancer [73].

## 4. Physical Cues Drive Cells Differentiation and Fate

In the last decades it became clear that cell behavior is far from being “controlled” by linear (digital) “commands” but rather by complex networks of molecular interactions and biophysical cues, spanning across different levels of structural and functional organization. Not only those interactions are context-dependent and thus cannot be understood by keeping cells in an inappropriate milieu, like that provided by 2D-cultures, but also they follow nonlinear dynamics which make impractical modeling processes with more than two variables. The switching between different stable states (representing differentiated or pathological phenotypes) requires that the activity/expression of several “signaling” molecules change in concert. Indeed, phenotype reversions are linked to the simultaneous coexpression of hundreds of different transcription factors and multiple downstream genes [74, 75]. To achieve a state-transition, no single point mutation is sufficient, and a cumulative effect linked to mutations will occur only if a critical state of the system as a whole is reached. It is worth of noting that the transition beyond that critical point “may be prevented or reversed by simultaneously manipulating a number of factors in the extracellular medium” [76]. Indeed, If multiple molecular elements must be tuned simultaneously to change cell phenotype, then it should be hypothesized that only a stimulus, outfitted with pleiotropic property, would perform that task, mainly based on stochastic fluctuations that enable transition from one attractor (phenotype) to another; that model may explain the genome-wide adaptability to environmental changes without requiring specific molecular signaling transducers [77] and why switching in between different cell fates can be triggered by changes in extracellular matrix structure, by inducing cell shape modification, and by adding aspecific chemical substances, electrical ion flows, and magnetic or gravitational fields [78–80]. Overall, those factors shared a meaningful property, given that they are able to modify the morphogenetic field and the biomechanical features of the systems [81].

Cells sense and respond to external physical forces and changes in matrix mechanics by modulating their endogenous cytoskeletal contractility. For instance, the mechanosensitivity of cells lies on the delicate force balance between the endogenous cytoskeletal contractility and external mechanical forces transmitted across the cell-ECM adhesions [82]. The force balance is transmitted across the mechanical continuum of ECM-integrin-CSK, which regulates integrin-mediated adhesion sites (such as FAK and Src signaling), providing the mechanical linkage between the ECM and the actin CSK. Exposure of cells to mechanical strain, fluid shear stress, or plating cells on substrates with varying elastic moduli activates integrins, which promote recruitment of scaffold and signaling proteins to strengthen adhesions and to transmit biochemical signals into the cell. These mechanotransduction pathways establish positive feedback loops in which integrin engagement activates actomyosin CSK contractility, which in turn reinforces adhesions. Thus, the level of CSK contractility generated inside the cell is directly proportional to the adhesion strength and the matrix elastic modulus and dictates their cellular responses [83]. Moreover, the way a cell senses and responds to a biochemical input mainly depends on the* physical state* of both cells and their microenvironment. For instance, TGF*β*-1 exerts a “dual” role on cancer cells, and that paradoxical behavior is well recognized as a challenging enigma that, still now, classic molecular biology has not been able to elucidate [84–87]. To the contrary, by referring data to a higher level of observation, that is, when the cell-microenvironment interaction or the tissue levels are kept in consideration, conflicting results end up as such, and paradoxes may likely find a compelling explanation. Indeed, soluble factors like TGF*β*-1 may trigger opposite outputs depending on the tissue stiffness; under mechanically unloaded conditions (floating matrices), TGF*β*-1 stimulated contraction directly as an agonist and indirectly by preactivating cells to express the myofibroblast phenotype, whereas, under mechanically loaded conditions (stressed matrices), TGF*β*-1 had no direct agonist effect on contraction [88].

Physical and biochemical changes occurring within the microenvironment are transmitted from the cytoskeleton to the nucleoskeleton, thus enabling the selective unfolding of chromatin [89]. The DNA is enveloped in histone proteins to form strand, further wrapped and folded. Gene switching (on/off) can proceed properly only if the appropriate section of chromatin is unpacked and exposed to the enzyme machinery. This physical rearrangement of the chromatin is mainly dependent on the tensional forces perceived by the cell-microenvironment system and further transmitted across the focal adhesion along the cytonucleoskeleton to the cell biochemical/genetic machinery. Therefore, different cytoskeleton arrangements end up in activating different gene sequences, leading to triggering different biochemical pathways [90]. The balance between tensional forces and the cytoskeleton architecture modulates thereupon several complex cell functions like apoptosis, differentiation, proliferation, and ECM remodeling among others. That model can help in understanding the “dual” role displayed by a lot of “signaling molecules,” selective sensitivity to drugs [91], and why cancer cell behavior may proceed regardless of their “mutated” genes [92]. That is precisely what means “to put the gene in a context,” given that cell responses to molecular “signals” tightly lie on the response of individual cells to mechanical tension and to the specific microenvironment in which cells are embedded. To date, an overwhelming body of data has revealed that mechanical tension generated through molecular interactions within the cytoskeleton is indeed critical for modulating molecular activity [93, 94] and to dramatically influence cell form and function [95]. In turn, interactions between epithelial cells and microenvironmental components (namely, stromal cells) change ECM composition as well as its biochemical-biophysical features [96].

Experimental results have provided compelling evidence of the key role played by the microenvironment in cancer initiation. Despite the presence of “growth factor,” normal cells cannot grow when they are free of adhesion to ECM [97], or if they are compressed into specific geometric space (i.e., only along a thin epithelial monolayer) [98]. Similarly, stimulated breast cancer cells cease to grow when are detached to their substrate in a microgravity field [99]. Therefore, an increase in “signaling molecules” alone cannot explain cell growth induction, given that physical interaction with the microenvironment enables cells to respond to soluble factors or genetic inputs. Even in autosomal dominant tumor predisposition syndromes, like neurofibromatosis-1 (NF-1), NF-1 inactivation results in increased astrocyte growth, but the augmented proliferation rate is actually unable to induce glioma formation [100]. To observe tumor formation in vivo, brain microglia carrying NF-1 heterozygosity are needed. In that model, microenvironmental components drive the epithelial transformation, mainly by providing disruption of ECM integrity (through the enhanced release of hyaluronidase) and subsequent activation of the MAPK-pathway. As expected, inhibition of hyaluronidase release or microglia activation dramatically reduces mouse optical glioma proliferation in vivo [101].

Overall, those results highlight how the microenvironment, mainly through its physical components, participates in promoting and shaping the carcinogenic process that can be considered as a “development gone awry” [102]. As recently recognized, “the physical laws and principles that define the behaviour of matter are essential for developing an understanding of the initiation and progression of cancer,” thus providing “opportunities for new insights into long-lasting problems in cancer research” [103]. This premise, well-grounded on experimental basis, represents another discontinuity point with respect to SMT which posits that “biological-information” carried out by genes constitutes the only (or the main) causative factor in driving cellular fate and behavior.

## 5. Microenvironment and Cancer: Methodological Issues

The term “microenvironment” encompasses discrete, interacting elements, such as extracellular matrix (ECM), stromal cells, molecular diffusible factors, configuration of the cell-stroma architecture [104], nonlocal control through field's forces [105], and topologic geometry of the emerging tissue [106]. In order to grasp such overwhelming complexity of interactions, we adopted a radical new perspective, which considers the interplay of both biochemical (proteomic, genetic, and metabolic) and biophysical (stiffness and surface tension) factors operating at different levels [107]. In other words, to understand tissue level phenomena, it is necessary to study the tissue and not single pathway in cells isolated from their tissue environment. The radical change in theoretical perspective requires a shift from the gene-centric paradigm to the cell-microenvironment system [108], a concept introduced as late as 1962 by Smithers [109], claiming a tissue-based “quality,” challenging the Boveri's prevailing view of cancer as a cell-based disease. This means that we must change the “level” of observation, by positioning ourselves where the process we are looking for really happens. Thus, “to understand the whole, one must study the whole” [110]; and if one wishes to study a tissue-based disease, like cancer, one must study the tissue.

This paradigmatic switch has important theoretical and methodological implications. First, cell function and behavior cannot longer be studied in isolation, that is, without taking into consideration their three-dimensional microenvironment. Two-dimensional cultures can be viewed for many aspects as true “artifacts,” which often makes them unreliable predictors of gene expression, tissue structure, cellular functions, and behaviour [111]. Moreover, also the actual response to many drugs is remarkably flawed by 2D-environment-based experiments [112]; instead, 3D cellular models have the potential to become a fundamental research tool in biology [113], given that they allow the integration of data generated by investigations carried out at different levels. This will result in models of tissues and organisms with enhanced predictive power [114].

Second, tissue and cytoskeleton/nucleoskeleton architecture, as well as mechanical forces (stiffness, shear stress [115], and surface tension), must be adequately weighted and investigated, a rather unusual request for a “traditional” biologist [116].

Third, molecular and genetic changes, involving both the epithelial and the stromal cells, should therefore be investigated in association and linked to the observed modification of the context.

Although much has been learned about molecular components and subcellular processes, the integration of data and models across a wide range of spatial and temporal scales, taking us from observations at the cellular or subcellular level to understand tissue level phenomena, remains an unchartered territory. Moreover, biophysical influences on cell behavior and differentiation can be adequately appreciated only by studying cells in their three-dimensional context and are therefore disregarded by current experimental methodologies almost fully based on 2D cultures. Overall, these considerations highlight another fundamental bias of modern biology, that is, the lack of a general theory for understanding biological organization. In order to cope with the increasingly appreciated complexity of living organism, implicitly, biologists have adopted a reductive approach, mainly based on a gene-centric paradigm, where causative processes are modelled according to a simplified, linear dynamics. However, reality is far more complex than the biochemical diagrams we are asked to trust. Biological complexity entails nonlinear dynamics, stochastic gene expression, interactions between biochemical and biophysical factors, and events acting simultaneously at different levels. From molecules to organs, levels are interrelated and interdependent, so that the organism is able to conserve and adapt the integrity of its structural and functional organization against a back-drop of continuous changes within the organism and its environment. That feature represents the updated interpretation of homeostasis, a concept formulated a century ago by W. Cannon and currently reinterpreted as autoconservation [117], functional stability [118], evolvability, or robustness [119]. Given that homeostasis is dramatically threatened or even disrupted in the course of several diseases, to understand such processes we are obligatory required to apply methodologies that explore nonlinear spatiotemporal systems with multiple levels of structural and functional organization. As pointedly discussed by Noble [120], one cannot understand the physiology or the pathology of cardiac rhythm by only referring to the gene expression and to the features of a single cardiomyocite. Similarly one cannot understand pathologic processes emerging at the cell-microenvironment level by only referring to “abstract” gene-regulatory circuits in the isolated cell.

## 6. Conclusions

The interaction between cells and their microenvironment, by involving both biochemical and biophysical cues, drives differentiating processes and contributes in a determinant fashion to cancer emergence. Considering cancer as a tissue-based phenomenon implies a profound rewiring of our experimental methodology, by requiring to move from cells and subcellular structures toward higher levels of organization. Namely, experiments should be undertaken in order to verify how to modify microenvironment biophysical features by means of chemical/pharmacological means in order to prevent or eventually to induce cancer reprogramming in 3D settings or on animal models.

Choosing between competing premises and testing alternative theoretical hypothesis have been the core component of the experimental science since the Renaissance. However, as Kuhn [121] has taught us, a widely accepted paradigm will hardly be dropped before a considerable amount of paradoxes and contradictions has been resolved. Such moment seems to have come. Indeed, the somatic mutation theory has eventually accumulated an impressive body of shortfalls and paradoxes, as admittedly claimed by its own supporters [2], as a cell-based approach can hardly explain the emergence of tissue-based processes, like cancer. However, as Niels Bohr once said “How wonderful that we have met with a paradox. Now we have some hope of making progress” [122]. Nowadays, that progress may be disclosed embracing new theoretical perspectives, taking the cells-microenvironment interplay as the privileged level of observation and assuming radically different premises as well as methodological frameworks [19, 123]. This may probably not be enough, and even new theories could prove to be incomplete. Yet, as once stated by Tzu thousands years ago, a new path, however long it may be, always begins with the first leap [124].
